# MAPSAR Image Simulation Based on L-band Polarimetric Data from the SAR-R99B Airborne Sensor (SIVAM System)

**DOI:** 10.3390/s90100102

**Published:** 2009-01-07

**Authors:** José Claudio Mura, Waldir Renato Paradella, Luciano Vieira Dutra, João Roberto dos Santos, Bernardo Friedrich Theodor Rudorff, Fernando Pellon de Miranda, Mario Marcos Quintino da Silva, Wagner Fernando da Silva

**Affiliations:** 1 National Institute for Space Research (INPE). São José dos Campos, 12227-010, São Paulo, Brazil; E-mails: mura@dpi.inpe.br; dutra@dpi.inpe.br; waldir@dsr.inpe.br; bernardo@dsr.inpe.br; jroberto@dsr.inpe.br; wagner@dsr.inpe.br; quintino@dss.inpe.br; 2 PETROBRAS (CENPES). Rio de Janeiro, Ilha do Fundão, 21941-598, Rio de Janeiro, Brazil; E-mail: fmiranda@petrobras.com.br

**Keywords:** SAR, MAPSAR simulation, SIVAM system, L-band radar, Amazon Region

## Abstract

This paper describes the methodology applied to generate simulated multipolarized L-band SAR images of the MAPSAR (Multi-Application Purpose SAR) satellite from the airborne SAR R99B sensor (SIVAM System). MAPSAR is a feasibility study conducted by INPE (National Institute for Space Research) and DLR (German Aerospace Center) targeting a satellite L-band SAR innovative mission for assessment, management and monitoring of natural resources. Examples of simulated products and their applications are briefly discussed.

## 1. Introduction

**Figure 1.**
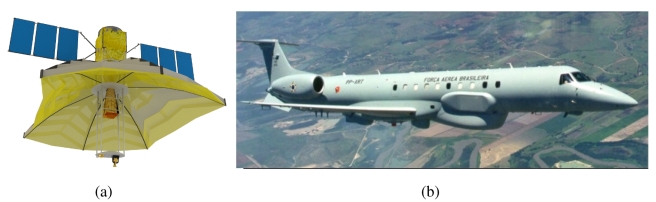
(a) MAPSAR satellite conception; (b) SAR-R99B on board of the EMB-145 aircraft.

Multi-Application Purpose SAR (MAPSAR) is a German-Brazilian feasibility study focusing on an L-band light SAR [1]. It is an innovative mission with an elliptical parabolic reflector SAR antenna based on INPE's Multi-Mission Platform (Figure 1a). It was conceived to provide a variety of L-band imaging modes, with polarimetry, interferometry, stereoscopy, and distinct viewing geometries, area coverage and resolution attributes. This kind of capability has normally been available to the remote sensing community on a number of airborne platforms, such as AIRSAR, E-SAR, CV-580, among others. Generally, with the exception of swath, aircraft systems produce SAR data with superior or similar quality than orbital imagery, providing a reasonable tool for evaluating the potential of a planned orbital system through simulations. At the end of the Pre-phase A it was agreed between INPE and DLR that a simulation campaign in Brazil would be important since the understanding with respect to application potential of polarimetric L-band was extremely limited and dependent on the limited number of data sets that have been acquired in the tropical conditions during the short-lived SIR-C/X-SAR missions. For the acquisition of L-band data in Brazil, the airborne sensor SAR-R99B from the Brazilian SIVAM (Amazon Surveillance System) is the most readily available and hence the sensor of choice for the MAPSAR simulation. The SAR-R99B was developed by MacDonald Dettwiler under a Raytheon Systems Corporation contract and is based on a modified EMBRAER (EMB-145) jet (Figure 1b), with multi-channel fully polarimetric SAR mapping mode attributes, cross-track Interferometric SAR modes, Spotlight SAR and Wide Area Surveillance with Ground Moving Target Indicator (GMTI) capability. To enable extensive mapping and monitoring missions in the Amazon region, 18 different mapping acquisitions and processing modes are available. Under the L-band strip map configuration, acquisitions are possible with 3, 6, or 18 m spatial resolutions and corresponding swathes of 20, 40, and 120 km. One, two or four channels of polarimetric data (HH, HV, VV, VH) may be collected. Simultaneous acquisition of the X-band (single strip map channel or channels of cross-track interferometric data) and L-band is also available with up to 6 channels of data being produced simultaneously [2].

Currently the MAPSAR Mission is in phase B. The work scope for this phase will be concluded after the complete system (satellite, ground and applications) preliminary design, including validation of all proposed solutions and make-or-buy decisions. A System Requirements Review (SRR) will be performed, showing that all performance requirements will be accomplished. Phase B conclusion is established for November, 2009, for a launch attempt by 2013. The joint venture will be carried out on 50/50% basis, both for WBS (Work Breakdown Structure) and cost sharing.

## 2. MAPSAR characteristics and simulation

MAPSAR is based on the use of a passive parabolic reflector antenna. Active planar phased array antennas with their electrical beam steering capability offer a high degree of flexibility to fulfill the user requirements. Therefore, they were proposed for missions like ALOS/PALSAR and TerraSAR-L. Nevertheless this antenna technology has significant drawbacks. It is quite heavy and the realization of high bandwidths and full polarimetric capability add additional complexity to the design. In addition, the weight of an antenna with this technology and the required size would exceed the capabilities of the Multi-Mission Platform. On the other hand, the concept of a parabolic reflector antenna offers several advantages. It is lighter, cheaper and the realization of a fully polarimetric system and the use of high bandwidths (for a high spatial resolution) are not at least as difficult as with an active phased array antenna concept. The drawbacks are a limited instantaneous swath width and strong limitations in electronic beam steering (no ScanSAR mode). User discussions showed that mosaicking of sub-swaths is acceptable for most applications as a compensation for the reduced instantaneous swath.

MAPSAR concept provides three different resolution modes (3 m, 10 m and 20 m) operated in the conventional stripmap mode. The chirp bandwidth is designed to the full 85 MHz frequency span endorsed by the World Administrative Radio Conference (WARC). This allows a high resolution (HR) mode on the order of 3 m, without increasing the complexity of the front-end and the antenna. For lower resolution modes the bandwidth is reduced accordingly. A single polarization mode (SPM), a dual polarization mode (DPM) and a quad polarization mode (QPM) are foreseen. For the low resolution (LR) mode the instantaneous swath width is limited in the near range of the access region by the antenna beam width. The high pulse bandwidth of MAPSAR makes it also very well suited for dual pass interferometry. This mode is of special interest for measuring biomass in combination with the full polarization capability (PolInSAR), to derive digital elevation models and surface deformation applications (DInSAR)

The main challenge of orbital imagery simulation using airborne data is related to the view geometry. For the same swath, the incidence angle variation across range is much higher than the spaceborne viewing case. Secondly, the signal power on the ground level is higher for the airborne case. Finally, the very distinct system bandwidth allows a higher spatial resolution with airborne. To overcome the incidence angle problem it is commonly necessary to acquire several strips of data with same look azimuth and use only a subset of each strip to produce a mosaic that could represent a spaceborne image with the intended incidence interval. Resolution is degraded and noise is added to attend to the spaceborne specifications.

**Figure 2.**
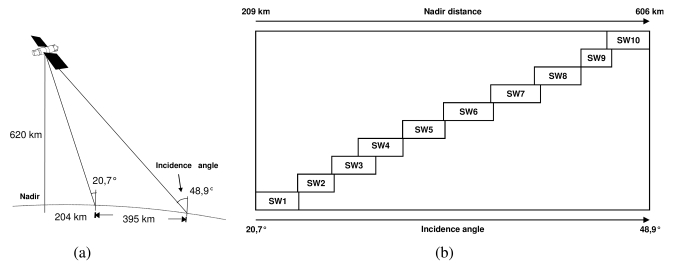
MAPSAR acquisition geometry (a) and beams for the Medium Resolution Mode (b). The simulated beams were the 9^th^ and the 10^th^.

Eight test-sites in Brazil (six in the Amazon region, two in the Northeast) were selected covering themes like Agriculture, Forestry, Geology and Mineral Exploration, Disaster Management, Costal Zone Studies, Geomorphology, Hydrology and Environmental Analysis. The sites were selected by potential end-users from governmental agencies, universities and private companies. Originally designed for surveillance and mapping purposes, the SAR-R99 sensor operates under high incidence range. Using the A1 Quad L+ X mode of SAR-R99B (incidence range: 39.6–71 degrees) it was only possible to simulate the MAPSAR Medium Resolution Mode, shown in Table 1 in bold format, particularly its far range viewing covered by beams 9 and 10 as shown in Table 2 and Figure 2b. It is important to mention that it was not possible to simulate polarimetric and interferometric conditions due to the impossibility of keeping the phase information in the whole mosaic, which was built from distinct time acquisition strips. In this way, only multi-polarized attributes were addressed.

**Figure 3.**
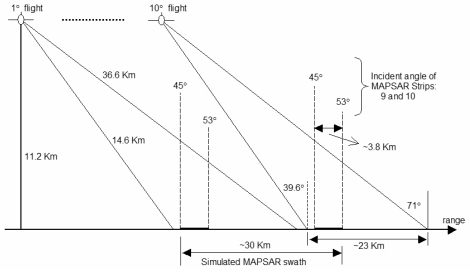
Flight geometry for the MAPSAR image simulation.

**Figure 4.**
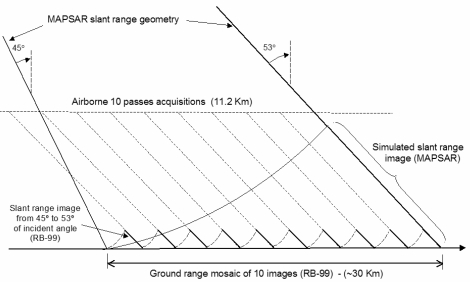
Slant range geometry for the spaceborne MAPSAR platform.

It is important to notice that the interval of incidence angle is slightly out of the proposed MAPSAR far range viewing. However, this selection was based on a compromise between a good signal/noise ratio for the SAR-R99B images and the number of flight tracks necessary to produce a simulated mosaic. Thus, the basic strategy for the simulation was to use a set of ten SAR-R99B scenes with a large overlap between them to produce a simulated MAPSAR image under a descending orbit (look-azimuth = 280 degrees). Strips from each image (1/10th), within an incidence angle interval of 45 to 53 degrees, were composed to create a simulated mosaic representing the MAPSAR Medium Resolution Mode Image, with spatial resolution of 10 m and swath width of 30 km (Figure 3). The ground range mosaic of each simulated scene was transformed to the slant range spaceborne geometry of the MAPSAR platform as depicted in the Figure 4. A total of 160 hours of flights was necessary to cover the test-sites during the simulation campaign carried out in 2005.

The simulated MAPSAR images represent multi-polarized data since only amplitude information related to L-HH, L-VV and L-HV was produced, even though the SAR-R99B images had been calibrated before the mosaic generation. Despite of the fact that the simulated products are composed by slices of the SAR-R99B images, caution was taken to avoid scalloping effect through the use of pattern antenna correction. This achieves a consistent tonal and contrast level across the mosaic such that the boundaries between adjacent strips were not easily seen.

## 3. Data processing

**Figure 5.**
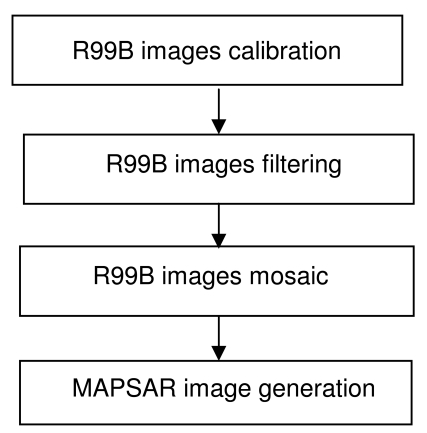
Processing flow to generate a simulated MAPSAR image

The processing steps to generate the MAPSAR simulated images are shown in Figure 5.

### 3.1. SAR-R99B image calibration imge generation mosaic

**Figure 6.**
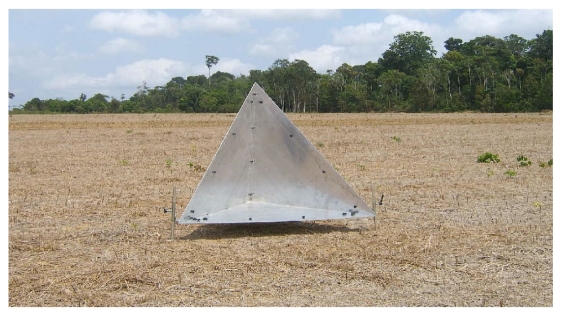
Corner reflector deployed in the Tapajós area.

During the data acquisition campaign 12 corner reflectors were deployed in two test areas: a) the first flown area in Coari (Amazon State) and b) the last flown area in Tapajós (Pará State). An example of a corner reflector deployment in the Tapajos area is shown in Figure 6.

The purpose of using corner reflectors for the first and last flown areas was to measure the polarimetric calibration parameters in the beginning and in the end of the campaign. The corner reflectors were use to determine the channel imbalance of the R99B SLC images, the cross-talk effect was removed based on the algorithm proposed by [3]. The corner reflectors were also used to generate the absolute calibrated products, the sigma naught images. The R99B SAR system can be considered as reciprocal, and under the general assumption of target reciprocity, HV and VH can be considered equal after calibration.

### 3.2. R99B image filtering

**Figure 7.**
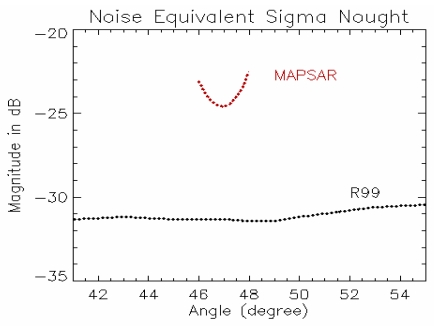
Plots of estimated NESN for the MAPSAR and the R99B system (HH polarization).

The MAPSAR and the R99B sensors have different characteristics concerning the system bandwidth, system noise and acquisition geometry since the first is a spaceborne sensor while the second is an airborne sensor. In order to simulate a MAPSAR image from the R99B images some constraint in terms of noise and bandwidth has to be applied for the R99B images. Corners reflectors were deployed nearby the Coari river and used as a reference to obtain the σ_o_ values based on the peak power method [4]. The noise equivalent sigma nought (NESN) of the R99B system was determined by using the backscatter signal related to the river. The NESN estimated profiles in relation to the incident angle variation of the HH polarization for the MAPSAR and the R99B sensors are shown in Figure 7. The curve for the NESN MAPSAR response can be explained by the antenna pattern variation within the incidence range. In order to calculate the MAPSAR NESN it was used the mean value of this curve for the selected incidence angle interval.

**Figure 8.**
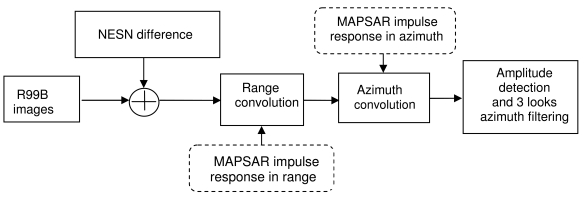
Processing flow of radiometric filtering to simulate the MAPSAR image.

The MAPSAR system will produce images that are noisier than those produced by the R99B system, therefore an amount of noise was added in the image of each polarimetric channel based on the NESN difference of each channel. Moreover, in order to accommodate the range and azimuth bandwidth to the MAPSAR characteristics, filters in range and azimuth directions were applied to the R99B images. Finally an appropriate average filtering was applied to the amplitude image to obtain the necessary three looks in the azimuth direction to attend the Medium Resolution Mode product characteristics as shown in Table 2. The processing steps are shown in Figure 8.

**Figure 9.**
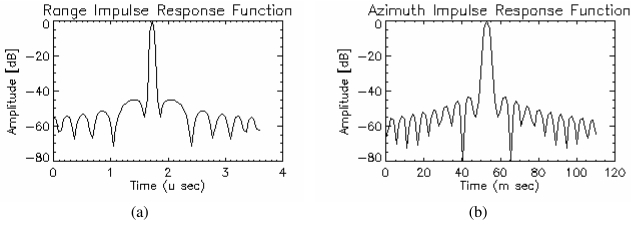
The MAPSAR Impulse Response Function for range (a) and azimuth (b).

The MAPSAR Impulse Response Function (IRF) for range (one look) and azimuth (three looks) with the Hamming window filters (α=0.54) are shown in the Figure 9 for the Medium Resolution Mode. The following parameters were used to build up these functions: range bandwidth = 21.25 MHz, sampling frequency = 22 MHz (complex data), azimuth bandwidth = 2100 Hz (middle swath), slant range distance = 898,276 m, platform velocity on the ground = 6,800 m/s and PRF = 2,680 Hz.

### 3.3. Mosaic of the SAR-R99 images

The mosaic generated for each test site area was composed of ten R99B multi-polarized geocoded images, based on the WGS84 ellipsoid, according to the scheme shown in the Figure 4. The state vector data of the R99B platform was used to geocode these images. The mosaic was performed for three polarizations, HH, VV and HV. During the mosaicking process care was taken regarding the radiometric continuity between the R99B geocoded images in the mosaic and the polarization response. Therefore, some parts in the mosaic presented small scalloping effect in areas of poor backscatter, such as water, basically due to the poor signal/noise ratio in the polarization HV in a range of incidence angle from 45° to 53° degree.

### 3.4. MAPSAR image generation

The last step of the simulation process was to transform the mosaic to the geometry of the MAPSAR spaceborne sensor. The mosaic data were projected to the MAPSAR slant range geometry and resampled to a resolution of 10 m in range and azimuth direction. The product provided to the user should represent a simulated L band multi-polarized image with three azimuth looks and spatial resolution of 10 m in range and azimuth directions, representing the Medium Resolution Mode (DPM) of MAPSAR, with a swath width of approximately 30 km, either in slant range and geocoded ground range versions. The slant-range images were delivered in Tiff format and the geocoded images in GeoTiff format (Projection: Lat/Long; Datum: WGS-84).

## 4. Examples of applications with simulated products

While many of the investigations using MAPSAR simulated data are still in progress, it is possible to generalize the findings to date, and apply these to plan future data usage from the orbital planned system. The MAPSAR simulation campaign provided the first opportunity for scientific studies of tropical target signatures using the SAR R99B multipolarized L-band with field campaigns carried out simultaneous with the flights. Three examples of the evaluation of MAPSAR products are briefly addressed: two in the Amazon Region (Tapajós National Forest and Solimões River) and one in the semi-arid environment (Luis Eduardo Magalhães municipality, Bahia State).

### 4.1. Land Cover/Use Classification (Tapajós National Forest, Pará State)

An assessment of using MAPSAR simulated images for land cover/use classification in the Tapajós National Forest, central part of the Brazilian Amazon, is presented in this section. This assessment was done using scenes over the test-site, acquired on September 12-13, 2005 with seven classes of general interest in the area (Figure 9c). Classification was performed by the ICM contextual classifier (1), based on Gaussian statistics [5]:

(1) $$g_{i}(x,\mu_{i})=-(\ln\left|\sum_{i}\right|+{\left(x-\mu_{i}\right)}^{T}\sum_{i}^{-1}(x-\mu_{i}))-\beta .\#\left\{t\in \partial_{x}:x_{t}=i\right\}$$

where g_i_ is the discriminating function for class i. In the first term of equation (1) *Σ_i_* and *μ_i_* are covariance matrix and the average vector for class i, respectively. The second term of equation (1) introduces the contextual information in the class assignment rule. *β* is called the attraction coefficient and it is estimated as described in [5]. *∂x* is a 3 × 3 neighborhood of x and *t* is a position (coordinates) inside this neighborhood. *β* is multiplied by number of the same class neighbors of the pixel being classified. A pixel is classified to a class i if g_i_ > g_j_, for all *i* ≠ *j*.

**Figure 10.**
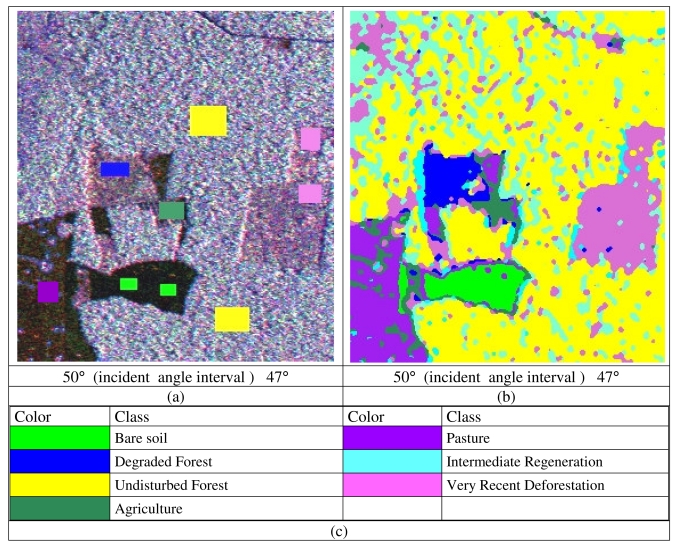
(a) RGB Color composition of the HH, VV e HV simulated MAPSAR images; (b) Classification map using all three polarization channels, (c) Land Cover/Use Classes.

The overall classification quality was considered satisfactory, with Kappa coefficient of agreement of 74% [5]. Particularly, it was possible to discriminate recent deforestation (where timber is still on ground) from primary forest. Also, degraded forest (under store is altered by fire or cleaning, but canopy is still up) could be discriminated from undisturbed forest. A previous study using JERS-1 HH polarization alone reports the difficulty for the discrimination of the recent deforestation [6] because known areas of forest and recent deforestation showed no difference in backscatter. Figure 10a presents the color composition of a small area near the Tapajós region indicating the training areas for the classes of interest and the respective color codes are presented in Figure 10c. Figure 10b presents the classification map where the most important classes for tropical forest monitoring studies - degraded forest, recent clear cut – could be easily distinguished from pasture, undisturbed forest and intermediate regeneration, using the three polarization channels.

### 4.2. Oil Spill Sensitivity Assessment (Solimões River, Amazon State)

Studies conducted by PETROBRAS (the Brazilian oil company) in the Central Amazon Region indicated a potential demand of 10 million cubic meters per day of natural gas to generate electric power in Manaus (capital of Amazonas State). These estimations encouraged PETROBRAS to build a pipeline that will transport in the near future six million cubic meters of natural gas per day from the Urucu River region to the Solimões Terminal (TESOL) in the vicinities of Coari City and from there to Manaus (under construction). In addition, about 50 thousand barrels per day of very light crude oil is transported by a petroleum tanker from TESOL to another terminal in Manaus, using the fluvial route of the Solimões River. Such an operation implies that there is potential risk of an oil spill affecting flooded forests along the alluvial plain. Containment strategies currently adopted by industry and governmental agencies include the definition of oil spill environmental sensitivity index (ESI) maps in Geographic Information Systems (GIS). The lowest ESI in Central Amazon is assigned to manmade structures (ESI 1) and the highest to aquatic vegetation banks (ESI 10a) and flooded forests (ESI 10b). A systematic monitoring using L-band radar images of water level changes beneath the forest canopy would provide priceless information for the construction of multitemporal sensitivity index maps to oil spills in different seasons of the Amazonian hydrological cycle [7]. SAR R-99B data were acquired on 01 June, 2005 and the study with simulated MAPSAR images has focused on improving information about oil spill environmental sensitivity in an area of the planned pipeline in the floodplain of Coari City. The investigation has encompassed: (1) results of Unsupervised Semivariogram Textural Classification (USTC) of high-flood season, from multi-polarized L-band simulated MAPSAR; (2) identification of flooded forest and flooded vegetation in different L-band polarizations, since these cover types are form the most oil-sensitive habitat in the region; and (3) analyses of the performance of USTC classification using statistical analysis related to each multi-polarized (HH, HV and VV) L-band image mosaic.

The USTC is a deterministic classifier, which provides the option of combining both textural and radiometric information [8]. Textural information is described by the shape and value of the circular semivariogram function, which has the following form:

(2) $$\gamma ({\text{x}}_{0},h)=(1/2n)\sum_{\theta =0.2\pi }(\text{DN}({\text{x}}_{0}+r)-\mu_{\text{H}}({\text{x}}_{0}))$$

where γ (x_0_,h) is the semivariogram function at pixel location x_0_ and radial lag distance of h pixels; DN(x_0_+**r**) is the digital number value at radial lag distance **r** from x_0_ (radius h, angle θ); μ_H_(x_0_) is the mean value of a circular neighborhood of radius H, center x_0_; H is the maximum radial lag distance (in pixels) suitable to describe the data; n is the number of pixel neighbors at radial lag distance h.

Textural information is also described by the digital number variance in a circular neighborhood of radius H around the pixel x_0_ (σ ^2^_H_(x_0_)). The DN variance is included in the classification procedure because it reflects the value of the semivariogram function for a very large lag distance (greater than H). For a pixel location x_0_ on the R99B image, the vector **Z**(x_0_), of dimension H+2, has the following form:

(3) $$Z(x_{0})=\left[{\text{DN}}_{\text{dsp}}(x_{0}),{\gamma (\text{x}}_{0,}1),\gamma ({\text{x}}_{0,}2),\ldots ,{\gamma (\text{x}}_{0,}\text{H}),{\sigma^{2}}_{\text{H}}({\text{x}}_{0})\right]s$$

The classification procedure was accomplished based on all components of this H+2 dimensional vector, calculated for each pixel location. The ISODATA clustering algorithm was applied in order to carry out the unsupervised classification of this set of vectors. After the unsupervised classification, results obtained from the clustering program were merged together through interactive class aggregation (an aggregate is a grouping of one or more classes considered to be of thematic significance).

**Figure 11.**
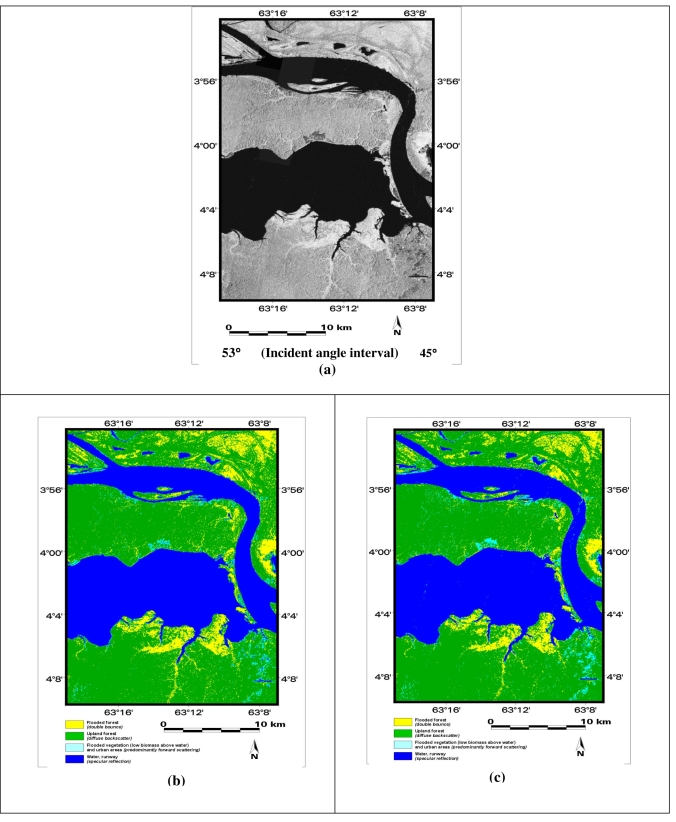
(a) Simulated LHH MAPSAR image; (b) USTC classification of simulated LHH MAPSAR image: pixels are labeled as water (blue) = 34.8%, flooded vegetation (cyan) = 2.4%, upland forest (green) = 54.1%, flooded forest (yellow) = 8.7%; and (c) USTC classification result of simulated LHH+LHV MAPSAR images: pixels are labeled as water (blue) = 34.7%, flooded vegetation (cyan) = 3.2%, upland forest (green) = 54.7 % and flooded forest (yellow) = 7.4%.

The statistical analysis for LHH, LHV and LVV USTC classification fully demonstrated that the LHH configuration yielded the best results for the individual mosaics. In order to further improve the proposed approach, the least correlated mosaics (LHH and LHV) were jointly processed. The combination of like (L-HH) and cross-polarized (L-HV) data presented better results if compared with the ones corresponding to the individual band as shown in Figure 11

### 4.3. Agriculture (Luis Eduardo Magalhães municipality, Bahia State)

**Figure 12.**
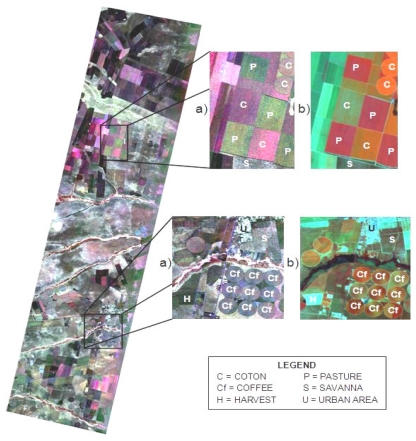
(a) A descending mode simulated MAPSAR geocoded image in color composition (VV-Red, HV-Green, HH-Blue) and (b) equivalent Landsat-5/Thematic Mapper (TM) image in color composition (band 4-Red, band 5-Green, band 3-blue) acquired on April 11, 2005.

Figure 12 illustrates the descending mode of a simulated MAPSAR geocoded image, acquired on 05 April 2005 in color composition (VV-Red, HV-Green, HH-Blue) over a quite intense cultivated region in the Luis Eduardo Magalhães municipality, western part of Bahia State.

This figure also shows parts of the equivalent Landsat-5/Thematic Mapper (TM) image in color composition (band 4-Red, band 5-Green, band 3-blue) acquired on 11 April 2005, which was used to perform a land use cover reference map for the study area. A visual comparison between the polarized backscatter from the simulated MAPSAR and the optical spectral reflectance from the Landsat-5/TM image indicates that they are somehow related. Some large and well developed crop fields of cotton, pasture, and coffee were selected within the SAR imaged portion of the study area.

In order to evaluate the simulated MAPSAR sensor image in terms of its capability to distinguish among different crops, mean backscatter values were calculated for single polarizations (HH, HV, VV) and combinations of two (VV-HH, HH-HV, VV-HV), and three (VV-HV-HH) polarizations in amplitude. These mean backscatter values were used to perform a cluster analysis based on the Chebychev distance and the weighted pair-group method (WPGM) [10]. Clusters were analyzed using dendograms and then classified according to the crop type predominance in each cluster. A confusion matrix was used to evaluate the classification result through the global accuracy (GA) and the Kappa index (k) [10]. Best result was obtained for the combination of three polarizations (VV-HV-HH), with GA = 85.4% and k = 0.804. The percentage of correctness for each crop was: 66.7% for cotton; 72.7% for Coffee-(X022A5) (Coffee Perpendicular – planting lines are mainly perpendicular to view direction) and 100% for both pasture and Coffee-II (Coffee Parallel – planting lines are mainly parallel to view direction), which indicates that MAPSAR images in fact present good potential to distinguish among agricultural crops.

## 5. Conclusions

The MAPSAR simulated images were evaluated for each test-site regarding the capability and potential for assessment, management and monitoring of natural resources in the Brazilian tropical environments, particularly in the Amazon region. Although the results are yet preliminary, they showed the potential benefits of the Mission and were important to support the decision of INPE-DLR to move to Phase B of MAPSAR [13]. Other recent results of the simulation assessment can be found in [11] and [12]. Further work will focus on the use of PALSAR data, under low incidence angles, to simulate MAPSAR QP mode.

## Figures and Tables

**Table 1.** Specification of MAPSAR imaging modes.

Parameters		Mode		High resolution SPM		Medium Resolution*DPM		Low resolution QPM	
Access region				near	far	near	**far**	near	far
spatial resolution	range (m) azimuth (m)			5.0 3.4	3.4 3.4	9.5 10.2	**9.9** **10.1**	20 20	20 20
Incidence (°)				19.73	47.22	20.74	**48.91**	20.8	36.13
Nominal Swath (km)				38.8	26.4	43.4	**43.0**	42.5	24.5
Chirp Bandwidth (MHz)				85	63.75	2×42.5	2×21.25	2×21.25	2×21.25
Looks number			range azimuth	1 1	1 1	1 **3**	**1** 3	1 6	1.56 6

* (Simulated MAPSAR mode)

**Table 2.** MAPSAR beams and incidence angles for the Medium Resolution Mode.

Beam	Incidence angle span (degrees)	Swath width (km)
1	20.74 - 24.63	43.4
2	24.52 - 27.67	36.8
3	27.47 - 31.07	44.3
4	29.66 - 33.2	45.1
5	33.16 - 36.32	42.7
6	36.2 - 39.67	50.2
7	39.49 - 42.7	50.1
8	42.32 – 45.13	46.9
9	45.13 - 46.87	31.0
10	46.6 - 48.91	43.0

**Table 3.** Confusion matrix for USTC classification of the different surface cover types (LHH+LHV).

PIXELS CLASS (HH+HV)	%	WATER	FLOODED VEGETATION	UPLAND FOREST	FLOODED FOREST
WATER		100	0	0	0
FLOODED					
VEGETATION		0	98.3	1.7	0
UPLAND FOREST		0	0	100	0
FLOODED FOREST		0	0	0	100
